# Management of Small Bowel Neuroendocrine Tumours: 10 Years’ Experience at a Tertiary Referral Centre

**DOI:** 10.3390/cancers15184438

**Published:** 2023-09-06

**Authors:** Ashley K. Clift, Panagiotis Drymousis, Alexander von Roon, Adam Humphries, Robert Goldin, Jamshed Bomanji, Sydney Leaman, Harpreet Wasan, Nagy Habib, Andrea Frilling

**Affiliations:** 1 Department of Surgery and Cancer, Imperial College London, Hammersmith Campus, Du Cane Road, London W12 0HS, UK; ashley.clift@phc.ox.ac.uk (A.K.C.);; 2 CRUK Oxford Centre, Department of Oncology, University of Oxford, Oxford OX1 2JD, UK; 3 Department of Surgery, Ealing Hospital, London North West University Healthcare NHS Trust, London HA1 3UJ, UK; 4 Department of Surgery, University College London Hospitals NHS Foundation Trust London, London NW1 2BU, UK; 5 Department of Gastroenterology, St Mark’s Hospital, London North West University Health Care NHS Trust, London HA1 3UJ, UK; 6 Department of Nuclear Medicine, University College London Hospitals NHS Foundation Trust London, London NW1 2BU, UK; 7 MRC Centre for Neurodevelopmental Disorders, King’s College London, London SE1 8WA, UK

**Keywords:** neuroendocrine, small intestine, surgery, multimodal treatment

## Abstract

**Simple Summary:**

Neuroendocrine tumours in the small bowel are often diagnosed when they are at an advanced stage. Treatment for these tumours can be a challenge, and there are multiple types of treatment available, such as surgery, medical options, and targeted radiotherapy. This study sought to report the outcomes of patients with small bowel neuroendocrine tumours treated at a specialist centre, where combined treatment strategies have been increasingly used.

**Abstract:**

Background: Neuroendocrine tumours (NET) arising from the small bowel are clinically challenging and are often diagnosed at advanced stages. Disease control with surgery alone can be demanding. Multimodal treatment concepts integrating surgery and non-surgical modalities could be of benefit. Method: Retrospective review of consecutive adult patients with SB NET treated at Imperial College Healthcare NHS Trust between 1 January 2010 and 31 December 2019. Data regarding clinicopathological characteristics, treatments, and disease trajectory were extracted and summarised. Overall and progression/recurrence-free survival were estimated at 5 and 10 years. Results: 154 patients were identified, with a median age of 64 years (range 33–87); 135/154 (87.7%) had stage III/IV disease at diagnosis. Surgery was used in 125 individuals (81.2%), typically with either segmental small bowel resection (60.8%) or right hemicolectomy (33.6%) and mesenteric lymphadenectomy for the primary tumour. Systemic and/or liver-directed therapies were used in 126 (81.8%); 60 (47.6%) had more than one line of non-surgical treatment. Median follow-up was 67.2 months (range 3.1–310.4); overall survival at 5 and 10 years was 91.0% (95% CI: 84.9–94.7%) and 82.5% (95% CI: 72.9–88.9%), respectively. Imaging-based median progression-free survival was 42.7 months (95% CI: 24.7 to 72.4); 5-year progression-free survival was 63.4% (95% CI: 55.0–70.6%); 10-year progression-free survival was 18.7% (95% CI: 12.4–26.1). Nineteen patients (12.3%) reached 10 years follow-up without disease recurrence and therefore were considered cured. Conclusions: Most patients with SB NET present in a metastasised stage. Multimodal treatment concepts may be associated with excellent clinical outcomes. Future work should explore optimal approaches to treatment sequencing and patient selection.

## 1. Introduction

Neuroendocrine tumours (NET) of the small bowel (SB), recently renamed under the umbrella term neuroendocrine neoplasms (NEN), present manifold clinical challenges: their incidence is steadily rising (0.2 per 100,000 in 1973 compared to 1.05 per 100,000 in 2012) [1,2], they are multifocal in 30–56% of cases [3,4,5], they frequently metastasise, and up to 70% of patients with SB NET are under-staged on morphologic imaging [6]. Somatostatin receptor (SSTR) based functional imaging, preferably in positron emission tomography (PET)/computed tomography (CT) techniques have evolved to become gold standard imaging tools since they yield significantly higher diagnostic sensitivity in the detection of both locoregional disease and distant metastases [7,8]. Whilst the vast majority of SB NET are well-differentiated grade (G) 1 or G2 lesions, approximately 90% still have mesenteric lymph node metastases and 45–70% have liver metastases at diagnosis [9,10,11].

Radical resection with an aim to eliminate the primary tumour/s including locoregional mesenteric metastases (and, if applicable, complete resection of liver metastases) is the cornerstone of efficient treatment, yielding long-term survival of patients with SB NET [11]. An analysis of the Surveillance, Epidemiology, and End Results (SEER) database v18 spanning the period 2000–2014 revealed a 5-year overall survival of 76% for SB NET patients who had any tumour-directed surgery, compared to 45.30% for those who were never treated surgically [12]. In the largest single-centre series to date on 603 SB NET patients who were diagnosed between 1985 and 2010 at Uppsala University, the 5-year overall survival was 67% [11]. Therein, locoregional resection was reported as a significant positive prognostic factor associated with increased survival compared to no surgery or explorative laparotomy only.

Complete resection of the primary tumour and attendant locoregional disease may be technically demanding [13]. Not only are the primary lesions frequently very small and difficult to identify even on gold standard imaging, but lymphadenectomy also presents challenges to surgery aiming to follow small intestine-sparing principles due to frequent encasement of vital mesenteric vascularity accompanied by desmoplastic reaction or mesenteric fibrosis [9,14]. In SB NET patients with liver metastases, gross bilobar multifocal spread accompanied by microscopic deposits is a common finding, reducing the chance of complete elimination of hepatic disease burden to less than 30% [11,15,16,17]. As treatment of metastatic disease occupies a pivotal role in the management of patients with SB NET, outcomes of recent landmark randomised controlled trials on somatostatin analogues (PROMID [18], CLARINET [19]), mTOR inhibitors (RADIANT IV [20]), and peptide receptor radionuclide therapy (NETTER-1 [21]) have contributed to the incremental implementation of multimodal treatment concepts combining surgery with non-surgical therapeutic strategies.

In this retrospective study, we report the outcomes of patients with SB NET in the era of multimodal treatment concepts and precision medicine in a tertiary referral centre and highlight the benefits and pitfalls of surgical approaches.

## 2. Materials and Methods

### 2.1. Study Population

A prospectively maintained database of consecutive patients with SB NET referred to the Imperial College Health Care NHS Trust (an ENETS Center of Excellence) between 1 January 2010 and 31 December 2019 was used for this retrospective case series study. Small bowel NET were identified as those with a primary tumour site from the duodeno-jejunal junction to the ileo-caecal junction. The diagnosis of a NEN was established histologically on surgical specimens or on biopsy; in most instances, this was on material from liver metastases. All but one patient had a follow-up of at least six months. All patients were discussed in local multidisciplinary team meetings. Patients consented to participation in clinical research. Regarding ethical approval, the institutional approval for the prospectively maintained patient database is under GEN_16; the collection and research use of integrated imaging, genomic, and other clinico-biological data is under 07/MRE09/54 (REC 22/WA/2836), and this specific study was approved by the HRA (IRAS ref: 35117, REC: 23/EM/0102).

### 2.2. Diagnostics

At baseline and during follow-up, a panel of diagnostic procedures including clinical assessment (e.g., investigations for carcinoid heart disease), imaging (cardiac ultrasonography, computed tomography (CT), CT colonography, magnetic resonance imaging [MRI], ^68^Gallium(^68^Ga)-DOTA-D-Phe1-Tyr3-Thr8-octreotide (DOTATATE) positron emission tomography (PET)/CT, and ^18^Fluorine-2-deoxyglucose (**^18^**F-FDG) PET/CT), endoscopic procedures (colonoscopy, video capsule endoscopy, and double balloon enteroscopy), and biochemical tests (serum gut hormones, chromogranin A and B, and 5-hydroxyindoleacetic acid (5-HIAA) in 24h urine) were used. The procedures were performed according to standard protocols as described previously [22].

### 2.3. Staging and Grading

Tumour staging and grading were according to the European Neuroendocrine Tumour Society (ENETS) tumour-node-metastasis (TNM) staging and grading system [23]. The World Health Organisation (WHO) classifications 2017 and 2019, respectively, were considered [24,25]. Liver metastases were considered synchronous if present at the time of primary tumour diagnosis and metachronous if diagnosed at least 6 months after primary tumour diagnosis.

### 2.4. Surgical Procedures

Emergency and elective cases were included, as were patients who were referred to us after incomplete/futile surgeries performed elsewhere. Our standard surgical approach included open laparotomy followed by systematic palpation of the entire small bowel starting at the Treitz ligament and ending at the caecal valve, assessment of mesenteric lymph node metastases involvement (stage I–IV) [26], and segmental small bowel resection/s including mesenteric loco-regional disease. A lymph node-first, intestinal-sparing principle was followed. For tumours localised in the terminal ileum, concomitant oncologic right hemicolectomy was performed. Some patients who had their initial surgery at our referring institutions had ileocecal resections. In recent years we have applied a modified approach in selected patients by starting the procedure laparoscopically, mobilising the bowel, and manually exploring the intestine after longitudinal enlargement of the port site incision for the camera and evisceration of all small bowel loops and mesentery. Cytoreduction of peritoneal carcinomatosis was applied as appropriate (peritoneal stripping and/or local electrocautery). In patients considered for liver surgery, the panel of procedures included resections according to Brisbane nomenclature [27], ≥ 70% resections (debulking), and segmental resections combined with intraoperative radiofrequency ablation (RFA). Liver resection was performed as an isolated measure or in combination with primary tumour resection. Selected patients with non-resectable liver metastases and no extrahepatic disease manifestation were evaluated for liver transplantation. Selected patients with advanced loco-regional disease were considered for multivisceral resections. In some cases, the tumour was discovered incidentally at the time of laparotomy for non-NET-related disorders. A patient with stage IV non-resectable mesenteric lymph node metastases and a multifocal primary tumour was investigated for multivisceral intestinal transplantation.

All patients considered for elective surgery were treated with octreotide (50 microgram/hour intravenously) for 12 h prior to surgery and 24 h thereafter to minimise the risk of carcinoid crisis. The definition of carcinoid crisis was according to criteria set by Fouche et al. [28]. Resected specimens were subjected to immuno-histochemical examination including assessment of Ki67% for tumour grading. Surgical morbidity (90 days) was assessed according to the Clavien–Dindo classification [29] and 90-day mortality was recorded.

### 2.5. Non-Surgical Treatments

Patients recommended treatment with somatostatin analogues were administered either 30 mg octreotide long-acting release (LAR) every 28 days, or 90 mg or 120 mg (preferably) lanreotide every 28 days. Those considered for mTOR inhibitors were prescribed 10 mg everolimus per day (starting dose). For high-grade NEN considered for chemotherapy, 5 fluorouracil (FU) and platinum-based regimens for poorly differentiated cancers were applied. Peptide receptor radionuclide therapy (PRRT) with Lutetium-177 (^177^Lu)-DOTA^0^-Try^3^-octreotate (^177^Lu-DOTATATE) (Lutathera^®^) followed protocols reported by us previously [30]. Selected patients were recruited for treatment in the NETTER-1 trial and the RADIANT IV trial. Liver-directed therapies included percutaneous CT or microbubble ultrasound-guided radiofrequency ablation (RFA), selective internal radiotherapy (protocol previously reported [31]), and, in the early phase of the study period, transarterial chemoembolisation (TACE).

### 2.6. Monitoring of Response to Treatment and Follow-Up

Regular blood tests for assessment of adverse events and imaging were carried out. Final restaging and assessment of response to treatment were performed with SSTR-PET/CT at 6 months after the last PRRT cycle. Responses were evaluated with the European Organisation for Research and Therapy of Cancer (EORTC) criteria [32] (PET component of PET/CT) as well as by the Response Evaluation Criteria in Solid Tumours (RECIST) [33] (CT component of PET/CT or MRI). Adverse events were assessed from laboratory data at the time of occurrence and graded according to the Common Terminology Criteria for Adverse Events (CTCAE) version 4.0 (NCI, Bethesda, Rockville, MD, USA). Patients were followed up in our clinic until death or study end date (31 December 2020). Follow-up encompassed standard biochemistry, assessment of blood and urinary standard tumour markers for NEN, morphologic imaging every 3–6 months, and SSTR-PET/CT-based imaging every 6–12 months or anytime earlier if suspicious findings on morphologic imaging were evident.

### 2.7. Statistical Analysis

Patient demographics and tumour characteristics were reported with descriptive statistics. Recurrence-free survival (RFS), progression-free survival (PFS), and overall survival (OS) were assessed by using the Kaplan–Meier methodology. Recurrence-free survival was calculated from the date of surgery to the date of disease recurrence evident on imaging. Progression-free survival was calculated from the date of initial diagnosis to the date of progression evident on imaging on first-line systemic treatment or liver-directed treatment. Overall survival was calculated from initial diagnosis to the date of death or the last follow-up visit, respectively. According to a statistical definition of cure previously described [34], patients alive and recurrence-free after 10 years were deemed cured. Log-rank tests were used to compare Kaplan–Meier curves, with *p* < 0.05 set as the threshold for significance.

We performed univariable and multivariable analyses of factors potentially associated with OS and PFS/RFS using Cox proportional hazards regression; however, these were purely exploratory due to the low number of events for most analyses. Stata V17 was used for all analyses.

## 3. Results

In total, 154 patients, of them 81 females, were included in the study (Table 1). The median age of the study cohort was 64 years (range: 33–87 years). One patient had hereditary multifocal small bowel NET (with multiple siblings affected). Two patients had more than one primary NET: one a metachronous pancreatic NET and the other a synchronous appendix NET. Both were negative on genetic screening for multiple endocrine neoplasia type 1 and 4, respectively.

Fifteen patients (9.7%) had the primary tumour resected in their local hospital prior to referral to our centre for further management. Tissue blocks of all of them were re-examined by a dedicated NET pathologist. Symptoms of carcinoid syndrome were present in 46 patients (29.9%). Various symptoms caused by local effect of the primary tumour such as pain, bleeding, and/or occlusion were recorded in 137/154 (89.0%). In 10/154 cases (6.5%), carcinoid heart disease was evident. In patients with the primary tumour still in place at the time of referral, the site of the primary tumour (small intestine) was identified on imaging and/or endoscopy in 87.1% (121/139). In total, 135 cases (87.5%) were in metastatic stage. Of them, 44 (28.6%) had nodal disease only, 2 (1.3%) had distant metastases only, and in 89 (57.8%) nodal disease and distant metastases were present. Liver metastases were evident in 88 patients (57.1%). The ‘classical’ tumour markers chromogranin A and 5-HIAA proved non-informative for confirmation of the diagnosis, disease stage, and/or follow-up. Eight patients (5.2%) had synchronous double malignancies, a NET, and an adenocarcinoma. The initial diagnosis of a NET was made in 13 patients (10.4%) at emergency laparotomy for intestinal obstruction or bleeding.

### 3.1. Surgical Treatment

Surgery for the primary tumour including loco-regional lymphadenectomy (and, in some cases, liver resection) took place in 125 patients (81.2%) (Table 2). In all patients with a unifocal tumour, the primary was in the ileum. Multifocal primary tumours were found in 46 cases (36.5%). The median number of tumours in multifocal cases was eight (range, 3–29). In all but two cases, multifocal primaries were missed on preoperative imaging. The number of primary tumours detected intraoperatively by surgeons corresponded with the numbers from the histologic analysis.

In the vast majority of cases, either segmental small bowel resection (60.8%) or right hemicolectomy (33.6%) was carried out. The grade of the primary tumour was G1 in 102 cases (81.6%), G2 in 22 cases (17.6%), and G3 in 1 case (0.8%). In cases of multifocal tumours with inter-tumoural grade variation, the highest grade was considered. Multivisceral resections were required in three cases to achieve an R0 situation. Thirteen patients (10.4%) underwent emergency laparotomy for intestinal obstruction caused by widespread metastases and/or peritoneal carcinomatosis. In four patients, a NET was discovered incidentally at the time of abdominal surgery for non-NET conditions. One patient had explorative laparotomy and biopsy only. In one patient with a multifocal ileal primary and extensive conventionally unresectable metastatic bulk in the mesentery, a radical resection followed by multivisceral liver-free intestinal transplantation took place after neoadjuvant peptide receptor radionuclide therapy (for more details see previous reports [35]).

In 90 patients (72.0%) locoregional lymph node metastases were present. Eighty-five patients (55.2%) and sixty patients (39.0%) had lymphovascular and perineural invasion, respectively. Complete resection of lymph node metastases was carried out in 84/90 (93.3%). In six patients with extensive mesenteric lymph node metastases (all also had liver metastases), only an incomplete resection of lymph nodes circumferentially encasing the mesenteric root (stage IV) was technically feasible without taking a high risk of postoperative short bowel syndrome. Two patients with an initial R0 resection of locoregionally limited disease had a reoperation for recurrence of mesenteric lymph node metastases after 12 months and 23 months, respectively.

Of patients with liver metastases, 18 (20.5%) (18/88 with LM) were considered as suitable for liver resection, either concomitant with primary tumour resection or as a separate procedure. All had type II liver metastases. Four patients with type II and type III liver metastases, respectively, were evaluated for liver transplantation. None passed the evaluation process as they did not meet the Milan criteria for liver transplantation of neuroendocrine liver metastases.

Five patients were referred to us after laparoscopic procedures with an intent to resect NET at hospitals elsewhere. In two, no tumour was found at the index procedure and in three only incomplete tumour resections were performed. The residual disease was evident on ^68^Ga-DOTATATE PET/CT performed for staging purposes after referral. All five patients underwent laparotomy and radical resection of primary tumours and locoregional lymph node metastases. Both patients in whom initially no tumour was identified had multifocal NET with 9 and 12 primary ileal tumours, respectively.

Two patients had cardiac surgery for advanced carcinoid heart disease. In both, multiple valve replacement was required. In one of them, right hemicolectomy, mesenteric lympadenectomy, and liver debulking followed cardiac surgery, and the other remained on systemic treatment with somatostatin analogues.

Postoperative 90-day morbidity was 5.6% (7/125); all were grade 1 according to the Clavien–Dindo classification. The 90-day mortality was 0%. No patient experienced a peri-operative carcinoid crisis.

### 3.2. Non-Surgical Treatment

Systemic and/or liver-directed therapies were administered to 125 patients (81.2%) during the study period, either as an adjunct to surgical treatment for control of residual non-resectable disease and/or for disease progression (the results are not presented in detail since they were published previously either as single centre series or within multicentre trials) [20,21,31]. In one of the patients, PRRT was used in the neoadjuvant setting prior to multivisceral transplantation. In 60/125 surgically treated patients (48.0%) more than one line of non-surgical treatment was required.

Liver-directed procedures included percutaneous RFA in 10 patients, SIRT in 31 patients, and TACE in 2 patients. Systemic targeted therapies encompassed somatostatin analogues (SSA), PRRT, and mTOR inhibitors in 89, 45, and 5 patients, respectively. Of the patients who had SSA as first-line systemic treatment, 64.1% (57/89) experienced disease progression within 24 months.

### 3.3. Follow-Up and Survival Outcomes

The median follow-up was 67.2 months (range, 3.1–310.4 months). No patient was lost to follow-up. At the last follow-up, 29 patients (18.8%) were alive with no evidence of NET, 102 (66.2%) were alive with NET, 18 (11.7%) died of NET, and 5 (all of them with stable disease, 3.3%) died of non-NET related causes.

**Figure 1:**
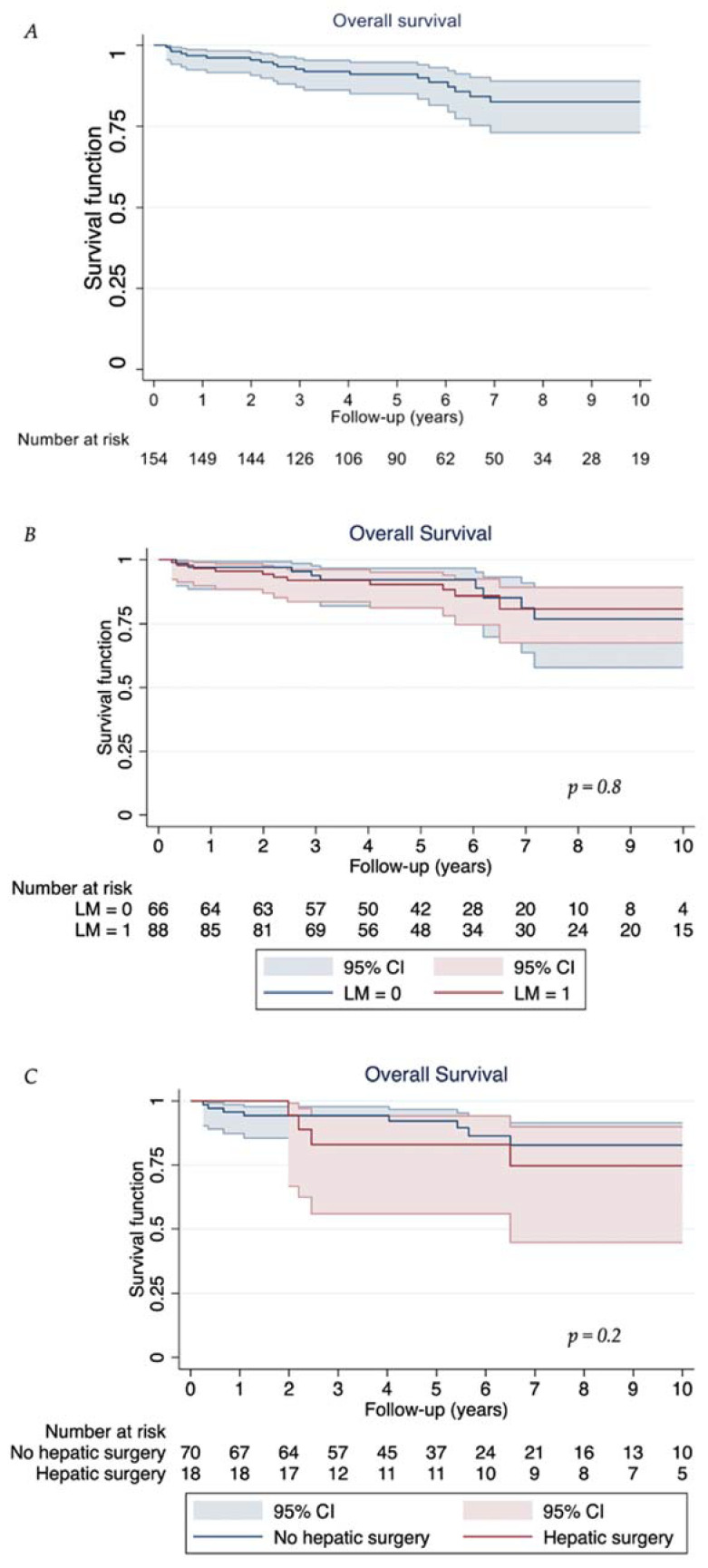
Overall survival for (**A**) (**top**) the study sample; (**B**) (**middle**) stratified by liver metastases status; (**C**) (**bottom**) whether or not patients with liver metastases underwent hepatic surgery. The *p*-values for parts (**B**,**C**) were obtained from a log-rank test.

The median overall survival was not reached. Overall survival at 1 year, 3 years, 5 years, and 10 years was 96.8% (95% CI: 92.4–98.5%), 92.6% (95% CI: 87.0–95.9%), 91.0% (95% CI: 84.9–94.7%), and 82.5% (95% CI: 72.9–88.9%), respectively (Table 3, Figure 1A). When discriminating between patients with no liver metastases and those with liver metastases, the 3-year, 5-year, and 10-year OS was 93.4% (95% CI: 83.3–97.5%), 91.6% (95% CI: 81.0–96.4%), and 79.8% (95% CI: 61.3–90.2%), respectively, and 92.1% (95% CI: 84.2–96.2%), 90.6% (95% CI: 82.0–95.2%), and 84.1% (95% CI: 72.1–91.2%), respectively (*p* = 0.8, Figure 1B). In those with liver metastases, there was no significant difference observed between those who underwent liver resection and those who did not (*p* = 0.2, Figure 1C).

Imaging-based median progression-free survival was 42.7 months (95% CI: 24.7 to 72.4 months). Progression-free survival at 1 year, 3 years, 5 years, and 10 years was 96.7% (95% CI: 92.4–98.6%), 84.3% (95 CI: 77.5–89.2%), 63.4% (95% CI: 55.0–70.6%), and 18.7% (95% CI: 12.4–26.1%), respectively. Recurrence-free survival was not calculated due to the small number of patients who developed recurrent disease (*n* = 2). The patient who underwent multivisceral transplantation was alive and tumour-free at 9 years following surgery [35]. Nineteen patients were (15.2%) alive and tumour-free at 10 years after surgery and were considered cured.

The univariate and multivariable analyses of factors associated with OS and PFS are reported in Table 4 and Table 5, respectively. The only significant associations (i.e., *p*-value < 0.05) identified were those for grade 2 tumours having reduced OS and PFS, but this must be interpreted in the context of the low numbers of events affecting precision and the extensive use of multiple lines of therapy.

## 4. Discussion

In this study comprising 154 consecutive patients with SB NET, we have demonstrated the following: (a) the vast majority of SB NET is present in stage III or IV although classified as low-grade neoplasia in 70% of cases; (b) despite high tumour stages and high hepatic tumour burden, radical surgery embedded within multimodal treatment concepts achieves favourable long-term outcomes with acceptable treatment-associated morbidity and nil mortality. The 5-year and 10-year OS of 91.0% and 82.5%, respectively, achieved in our cohort support the notion that cytoreductive surgery should be considered in all SB NET patients fit for surgical intervention [36,37,38]. Of note, a cure was likely achieved in 15%. Overall survival did not differ between patients with liver metastases and those with no hepatic involvement. Overlapping confidence intervals of the two groups likely reflect heavy use of successful, continued lines of systemic and/or liver-directed treatment in combination with surgery. No ‘predictors’ of survival were identified—we hypothesise that multimodal treatment approaches contributed to the favourable long-term survival in this series compared with the 5-year survival (all stages) in the range between 53% and 63% noted in earlier reports [39,40] and the 5-year and 10-year OS of 67% and 37%, respectively, reported in a systematic review in patients in metastasised stages [41].

The outcome of randomised controlled trials for systemic treatment of SBNET, PROMID [18], CLARINET [19], RADIANT IV [20], and NETTER-1 [42], which all failed to show impact on OS, further underpins the pivotal role of surgery.

When planning surgery for SB NET, several issues should be considered. Preoperative imaging frequently understages the disease, both in terms of the primary tumour and the metastases. In our series, 36.5% had multifocal primaries that were only detectable in their full extent by meticulous intra-operative palpation. In the series reported by Pasquer et al. [10], multicentric primaries were diagnosed in 33% and missed in 61% on preoperative imaging. Keck et al. found multifocal primaries in 56% of them, with 72% located within 100 cm of the ileocolic valve [3]. Clinical behaviour and survival are comparable between multifocal tumours and unifocal counterparts [4,5]. Based on the results of complete genome sequencing of tumours from patients with multicentric NET, Elias et al. suggested an independent clonal origin of such lesions [43].

Other underestimated findings on preoperative imaging are mesenteric tumour deposits and peritoneal carcinomatosis. The latter was observed at laparotomy in 12.8% of our patients and in 20–30% in other series [11,44]. Fata et al. [45] found mesenteric tumour deposits described as discrete but irregular mesenteric tumour nodules frequently located adjacent to neurovascular bundles and discontinuous from the primary neoplasm in 68% of 132 resected small bowel NET. Since both peritoneal carcinomatosis and mesenteric tumour deposits have been reported as independent factors of prognostic relevance, they should be considered when planning cytoreduction [10,11,44,45].

In contrast to NET originating from the pancreas or lung, high-grade NET or neuroendocrine carcinoma (NEC) are rarely seen in the small intestine. In line with observations made by others, the majority of our patients had G1 or G2 with low Ki67 tumours [11,36,45]. When assessing the Ki67 index, intra-tumoural and inter-tumoural grade heterogeneity should be considered. In our previous study, we have shown discrepancies in grading between primary gastroenteropancreatic NET and their metastatic sites in 35.3% of cases [46]. In 77.8% of patients with liver metastases, the Ki67 % was higher in liver lesions than in the primary tumour.

An open approach as recommended in current guidelines [13,47] is still considered to be a gold standard since it facilitates extensive manual palpation of the entire small intestine, secure access to lymph node metastases at the root of the mesentery, and synchronous liver debulking in selected cases. Laparoscopic resection is feasible [48,49]; however, solid evidence for the true benefit for the patient is still lacking. Conversion rates of 30% [50] and as high as 80% in patients with stage III or stage IV mesenteric lymph node metastases [51] have been reported.

Abdominal lymph node metastases are present in 75–90% of SB NET at initial diagnosis [9,10,52]. Their complete resection is pivotal for favourable long-term survival; however, this is challenging when there is an encasement of the mesenteric vessel origin and/or retropancreatic space, particularly in the presence of retractile fibrotic mesenteritis and/or desmoplastic reaction [11,53]. An optimal lympadenectomy should comprise at least eight lymph nodes under consideration of limited resection of the small intestine and utilisation of intestinal sparing techniques [54,55,56]. In patients with inadequate primary resection, a reintervention irrespective of negative imaging results should be considered [57]. Although 72% of patients in our series had lymph node metastases, 93.3% of these had them completely resected, and only two showed evidence of recurrence in the lymph nodes thereafter. This is in contrast to the published experience in patients with neuroendocrine liver metastases, which recur in virtually all cases [58].

The present study has limitations. The study sample comprises those treated at a tertiary referral centre; this may affect the transportability of findings to other settings, such as the high incidence of distantly metastatic disease in this group, and that all patients underwent elective surgery. Further, due to being managed in a tertiary centre, all patients underwent 68GA DOTATATE PET/CT prior to treatment decisions, which could have increased disease detection (e.g., identifying deposits that could have been ‘missed’ by other imaging modalities) and therefore exerted influence on treatment selection. The retrospective nature of this case series is also unable to ascertain the effects of specific treatment decisions on outcomes.

A substantial number of reports has shown that hepatic resection for neuroendocrine liver metastases provides favourable outcome with 5-year overall survival rates of 60–100% [15,16,38,59]. As shown in this series and in the experience of others, only about 30% are good candidates for hepatic resection (< 45% liver involvement, ≥ 70% cytoreduction, resectable/limited extrahepatic spread). In a systematic review and meta-analysis of outcomes of liver resection in patients with stage IV pancreatic NET, the median 1-year, 3-year, and 5-year overall survival in the resected group was 92.7%, 76.9%, and 67.5%, respectively, compared with 77.3%, 40.9%, and 26.6%, respectively, in the non-resected group [60]. As the authors rightly highlight, these data must be taken with caution since there were no randomised controlled trials, the selection for patients was biased by their suitability for surgery, and the treatment in the no liver-resection group was heterogeneous, ranging from primary tumour resection as the only measure to multimodal treatment concepts. Not achieving hepatic resection was the only independent factor of survival in the retrospective series in patients with SB NET and synchronous liver metastases reported by Addeo et al. [61].

In the light of high recurrence rates [62,63,64] and apparently no difference in the long-term survival outcome between patients who had R0 liver resection and those with R1 or R2 status [65,66], a shift from classical major resections [67] to more parenchyma preserving procedures [66,68] and acceptance of a debulking threshold of 70% in contrast to previous 90% has evolved over the last decade. In the series of Scott et al. encompassing 184 patients who had 188 cytoreductive surgeries, the median overall survival and progression free-survival was 89.4 months and 22.5 months, respectively [69]. In addition, 70% debulking was associated with better OS compared with < 70%, and 90% cytoreductive surgery was not associated with improved OS when compared with 70–90%.

The appropriateness of primary tumour resection in asymptomatic SB NET patients with non-resectable liver metastases is debated controversially. While proponents argue that primary tumour resection avoids local complications and prolongs survival, opponents suggest resection in this setting only if symptoms occur. The results of a systematic review were in favour of a proactive approach; median overall survival was 112 months in the primary tumour resection group compared with 60 months in the conservatively managed group. Five-year overall survival rates were 74% and 44%, respectively [70]. A study performed by Bennet et al. demonstrated that upfront small bowel resection was associated with lower rates of readmission and intervention compared to non-operative management [71]. In a single-centre series reported by Daskalakis et al., prophylactic upfront locoregional surgery had no survival advantage [72]. Moreover, delayed surgery was associated with fewer laparotomies for intestinal obstruction. Of note, emergency procedures were included in the latter group. In the experience of Ahmed et al. [15], in contrast, primary tumour resection and the Ki67 index were the only independent predictors of survival in the group of 360 patients with SBNET metastasised to the liver. Results reported by Kaemmerer et al. in patients with stage IV SBNET considered for peptide receptor radionuclide therapy supported upfront primary tumour resection leading to a median overall survival and progression-free survival of 134 months and 18 months, respectively, compared to 67 months and 18 months, respectively [73].

Frequently metastatic disease, high recurrence rates after hepatic resections, and promising results of systemic medical therapies and interventional liver directed procedures call for the consideration of multimodal concepts (combined surgical and non-surgical modalities) in the management of SB NET. In this series, multimodal treatment was applied in more than two third of patients. Of them, 87% had somatostatin receptor targeted therapy. Although in NEN, a neoadjuvant approach utilising peptide receptor radionuclide therapy has been mainly used in those originating from the pancreas [74], results in small bowel NET have been shown in few reports [30,75,76]. The recent case series of Fisher and colleagues comprised 17 patients with metastatic ileal NET and found a commendable 93% 5-year overall survival, and a 70% progression-free survival with the use of SSAs after surgery [76].

Genomic, transcriptomic, metabonomic, and epigenetic studies of SB NET have contributed to the delineation of novel molecular subgroups with differing risks of metastases and aggressive behaviour [77,78,79]. Future avenues of research should focus on the selection of treatment based on individual patient and tumour characteristics, optimal sequence of treatment modalities, and effective monitoring of treatment response utilising molecular-based markers [80,81]. 

## 5. Conclusions

Multimodal treatment strategies may be associated with excellent outcomes in SBNET, even though patients often present in advanced stages of the disease. Future study in this tumour type should focus on ascertaining the optimal sequencing and combinations of different therapies and explore their impact on overall survival.

## Figures and Tables

**Table 1:** Demographic and clinicopathological characteristics of the study cohort. The stage is as per the AJCC staging system; the grade is as per the ENETS/WHO grading system.

Parameter	*N* (Column % unless Otherwise Specified)
Total patients	154
Age at initial diagnosis	Median 64 years (range 33–87)
<50 years	24
50 to 59 years	35
60 to 69 years	50
70 to 74 years	21
75+ years	24
Sex	
Female	81 (52.6)
Male	73 (47.4)
Stage at initial diagnosis (on imaging)	
Stage I/II—localised disease only	19 (12.3)
Stage III—nodal metastases only	44 (28.6)
Stage IV—distant metastases (with or without nodal metastases)	91 (59.1)
Tumour grade	
G1	107 (69.5)
G2	35 (22.7)
G3	1 (0.7)
Not available	11 (7.1)
Site of distant metastases	
Liver only	70 (45.5)
Liver + bone	7 (4.5)
Liver + mesenteric mass	3 (2.0)
Liver + peritoneum or liver + omentum	4 (2.6)
Peritoneum + bone	2 (1.3)
Mesenteric mass	2 (1.3)
Bone only	2 (1.3)
Liver + lung + bone	1 (0.6)
Primary tumour location	
Ileum	154 (100)
Jejunum (all multifocal, also lesions in ileum)	3 (1.9)
Carcinoid syndrome present	46 (29.9)
Carcinoid heart disease present	10 (6.5)
Surgery used as first line treatment	125 (81.2)
Non-surgical treatment use (at least once during clinical course)	
Somatostatin analogues	89 (57.8)
Peptide receptor radionuclide therapy	45 (29.2)
Selective internal radiotherapy	31 (20.1)
Radiofrequency ablation	10 (6.5)
mTOR inhibitor	5 (3.3)
Transarterial chemoembolisation	2 (1.3)

**Table 2:** Surgical and histological characteristics of patients with small bowel neuroendocrine tumours. All individuals undergoing emergency surgery did so prior to referral to the tertiary centre. ‘Not available’ for pT staging refers to the unavailability of the full pathological report of surgical specimens at data extraction. * Surgery was performed at referring hospitals (while the pathologist at our centre re-reviewed the histopathology slides to confirm the diagnosis of well-differentiated NET, there was insufficient tissue remaining for Ki67 staining).

Parameter	*N* (Column % unless Otherwise Specified)
Total patients undergoing surgical treatment	125
Type of surgery	
Small bowel resection	76 (49.4)
Right hemicolectomy	42 (27.3)
Multivisceral resection	3 (2.0)
Incidental finding during other abdominal surgery	4 (2.6)
Resection + modified multivisceral transplantation	1 (0.7)
Mesenteric lymphadenectomy (complete)	84 (67.2)
Mesenteric lymphadenectomy (incomplete)	2 (1.4)
Repeat lymphadenectomy after lymph node recurrence	2 (1.4)
Emergency surgery	
Emergency index surgery	13 (10.4)
Exploratory laparotomy and biopsy only	1 (1.4)
Tumour grade (as per ENETS-WHO system)	
G1	92 (73.6)
G1	28 (22.4)
G3	1 (0.8)
Not available *	4 (3.2)
Primary tumour stage (as per ENETS-UICC system)	
pT1	3 (2.4)
pT2	15 (12.0)
pT3	26 (20.8)
pT4	42 (33.6)
Not available	39 (31.2)
Lymph node metastases	
N0	35 (28.0)
N1	90 (72.0)
Multifocal primary tumour	46 (36.8)
Mean number of lymph nodes resected per patient	18 (range 7–46)
Mesenteric tumour deposits present	6 (4.8)
Perineural invasion present	60 (48.0)
Lympho-angioinvasion present	85 (68.0)
90-days surgical morbidity	7 (5.6)
90-days surgical mortality	0 (0)

**Table 3:** Kaplan–Meier estimates of survival functions at 1 year and 3, 5, and 10 years after initial diagnosis.

Overall Survival	Kaplan–Meier Estimate (95% CI)
1-year	96.8% (92.4 to 98.5)
3-year	92.6% (87.1 to 95.9)
5-year	91.0% (85.0 to 94.7)
10-year	82.5% (73.0 to 88.9)
**Progression-free survival**	
1-year	96.7% (92.4 to 98.6)
3-year	84.3% (77.5 to 89.2)
5-year	63.4% (55.0 to 70.6)
10-year	18.7% (12.4 to 26.1)

**Table 4:** Associations between selected clinicopathological characteristics and 10-year overall survival were assessed using hazard ratios estimated using Cox proportional hazards modelling. Not estimable: non-convergence of the Cox proportional hazards model (small cell counts). N/A: not applicable.

Univariable			Multivariable		
Parameter	Hazard Ratio (95% CI)	*p*-Value	Parameter	Hazard Ratio (95% CI)	*p*-Value
Age (per year)	1.04 (1.00 to 1.08)	0.038	Age (per year)	1.04 (1.00 to 1.10)	0.099
Sex (female vs. male)	1.05 (0.43 to 2.59)	0.916	Sex (female vs. male)	0.77 (0.29 to 2.02)	0.591
Stage			Stage		
Stage I/II	1 (reference)		Stage I/II	1 (reference)	
Stage III	1.24 (0.25 to 6.14)	0.796	Stage III	0.65 (0.11 to 3.82)	0.631
Stage IV	1.20 (0.29 to 5.40)	0.808	Stage IV	0.53 (0.09 to 3.26)	0.497
Grade			Grade		
1	1 (reference)		1	1 (reference)	
2	2.79 (1.08 to 6.75)	0.033	2	3.22 (1.20 to 8.62)	0.020
3	Not estimable	N/A	3	Not estimable	N/A
Multifocal primary			Multifocal primary		
No	1 (reference)		No	1 (reference)	
Yes	1.15 (0.44 to 2.99)	0.779	Yes	1.03 (0.35 to 2.97)	0.959
Carcinoid syndrome			Carcinoid syndrome		
No	1 (reference)		No	1 (reference)	
Yes	0.93 (0.36 to 2.40)	1.00	Yes	1.03 (0.35 to 2.99)	0.958
Surgery as 1st line treatment			Surgery as 1st line treatment		
No	1 (reference)		No	1 (reference)	
Yes	0.93 (0.31 to 2.77)	0.897	Yes	0.57 (0.13 to 2.47)	0.454
Lymphovascular invasion	0.94 (0.30 to 2.80)	0.899	Lymphovascular invasion	Not estimable	N/A
Perineural invasion	1.74 (0.72 to 4.19)	0.215	Perineural invasion	2.25 (0.69 to 7.29)	0.177

**Table 5:** Associations between selected clinicopathological characteristics and 10-year progression-free survival were assessed using hazard ratios estimated using Cox proportional hazards modelling. Not estimable: non-convergence of the Cox proportional hazards model (small cell counts). N/A: not applicable.

Univariable			Multivariable		
Parameter	Hazard Ratio (95% CI)	*p*-Value	Parameter	Hazard Ratio (95% CI)	*p*-Value
Age (per year)	1.00 (1.00 to 1.02)	0.188	Age (per year)	1.00 (0.99 to 1.02)	0.369
Sex (female vs. male)	1.26 (0.88 to 1.88)	0.205	Sex (female vs. male)	1.03 (0.69 to 1.54)	0.867
Stage			Stage		
Stage I/II	1 (reference)		Stage I/II	1 (reference)	
Stage III	1.17 (0.59 to 2.33)	0.656	Stage III	1.10 (0.53 to 2.33)	0.785
Stage IV	1.32 (0.70 to 2.49)	0.389	Stage IV	1.32 (0.63 to 2.77)	0.464
Grade			Grade		
1	1 (reference)		1	1 (reference)	
2	1.61 (1.07 to 2.43)	0.024	2	1.71 (1.10 to 2.67)	0.018
3	1.17 (0.16 to 8.48)	0.873	3	1.69 (0.22 to 13.18)	0.615
Multifocal primary			Multifocal primary		
No	1 (reference)		No	1 (reference)	
Yes	1.32 (0.89 to 1.97)	0.159	Yes	1.37 (0.90 to 2.08)	0.146
Carcinoid syndrome			Carcinoid syndrome		
No	1 (reference)		No	1 (reference)	
Yes	1.07 (0.72 to 1.56)	0.743	Yes	0.73 (0.45 to 1.17)	0.190
Surgery as 1st line treatment			Surgery as 1st line treatment		
No	1 (reference)		No	1 (reference)	
Yes	0.87 (0.56 to 1.34)	0.519	Yes	0.95 (0.54 to 1.69)	0.865
Lymphovascular invasion	0.80 (0.51 to 1.37)	0.500	Lymphovascular invasion	Not estimable	N/A
Perineural invasion	0.95 (0.65 to 1.41)	0.815	Perineural invasion	0.95 (0.59 to 1.52)	

## Data Availability

Due to the nature of the clinical data, information governance considerations, and the remit of the consent gained from individual participants for inclusion in the institutional research database, the raw data used for this study cannot be made available to other investigators.
